# The impact of heart valve replacement surgery start time on patients with rheumatic heart disease

**DOI:** 10.3389/fcvm.2026.1693694

**Published:** 2026-02-13

**Authors:** Chonggang Wang, Songsong Zhang, Mei Li, Wenbin Zhang

**Affiliations:** 1 Department of Cardiac Surgery, Guizhou Provincial People’s Hospital, Guiyang, Guizhou, China; 2 Department of Intensive Care Unit, Guizhou Provincial People’s Hospital, Guiyang, Guizhou, China

**Keywords:** rheumatic heart disease, valve surgery, operative timing, chronobiology, outcomes

## Abstract

**Objectives:**

Rheumatic heart disease (RHD) remains prevalent in developing regions, often leading to significant valvular damage. This study aimed to assess whether the start time of heart valve replacement surgery influences short-term clinical outcomes in patients with rheumatic heart disease.

**Methods:**

This retrospective analysis included 300 adult patients who underwent elective valve replacement surgery between January 2020 and January 2023. Patients were divided into two groups based on surgery start time: morning (AM group: 7 a.m.–1 p.m.) and afternoon (PM group: after 1 p.m.). Propensity score matching and logistic regression were used to compare mortality, complications, intensive care unit (ICU) stay, and hospitalization.

**Results:**

After matching, 283 patients were analyzed (AM: 137, PM: 146). No significant differences were found between groups in postoperative mortality (odds ratios = 0.31, *P* = 0.17), bleeding reoperation (*P* = 0.70), blood product usage (*P* > 0.60), ICU stay (*P* = 0.12), ventilation time (*P* = 0.37), or hospitalization cost (*P* = 0.40). However, total hospitalization time was longer in the AM group (*P* = 0.01), influenced by factors such as cardiac function, liver insufficiency, and surgical complexity.

**Conclusions:**

Surgery start time did not significantly affect short-term outcomes in patients with rheumatic heart disease undergoing valve replacement. Differences in hospital stay duration may be attributable to patient-specific factors and surgical complexity.

## 1 Introduction

Rheumatic heart disease (RHD) has been nearly eradicated in developed nations; however, it continues to impose a significant public health burden across many developing regions, including China (1). Its hallmark pathological feature is damage to the heart valves, particularly the left heart valve, which serves as a common indicator of the disease (2). This damage is frequently associated with left ventricular myocardial fibrosis, resulting in progressive deterioration of cardiac function (3). For patients with severely damaged valves, heart valve repair or replacement is widely accepted as the primary treatment approach (4).

Complex cardiac surgeries often require collaborative efforts, both in the operating room and in the postoperative intensive care unit. In countries with large populations and high patient volumes, it is common for patients to undergo procedures in the afternoon. However, afternoon surgeries may present unique challenges, including surgeon fatigue due to prolonged work hours. Moreover, surgeries performed in the afternoon may result in postoperative care occurring outside standard working hours, potentially leading to decreased staffing and diminished vigilance during nighttime monitoring. While the impact of chronobiology on cardiovascular regulation is increasingly recognized, its specific influence on patients with rheumatic heart disease undergoing valve replacement remains poorly understood (5).

A previous study demonstrated that patients with aortic stenosis who underwent aortic valve replacement (AVR) surgery in the afternoon experienced lower rates of major adverse cardiac events than those who underwent the procedure in the morning (6). Subsequent research with larger sample sizes concluded that the timing of AVR surgery did not significantly affect clinical outcomes (7, 8). However, the study focused on AVR surgery alone and did not include patients with rheumatic heart disease. To date, no specific study has examined the postoperative impact of the start time of valve replacement surgery on patients with rheumatic heart disease. Given the unique impact of rheumatic heart disease on heart structure, it is crucial to consider whether selecting the optimal start time for surgery could be more advantageous for patients. Accordingly, this study aimed to evaluate whether the start time of valve replacement surgery influences the incidence of major adverse cardiac events and early postoperative survival outcomes in patients with rheumatic heart disease.

## 2 Methods

### 2.1 Study sample

This retrospective cohort study included the data of all adult patients who were hospitalized at our facility between January 2020 and January 2023 with a confirmed diagnosis of rheumatic heart disease who underwent valve surgery. Patients were divided into two different treatment groups. The operating surgeons and the attending doctors had equivalent qualifications and comparable years of professional experience. All surgical procedures adhered to the definitions of the Society of Thoracic Surgery (STS), and no absolute contraindications were present. Patients who underwent surgery during the same hospitalization to reduce the chance of further clinical deterioration, (emergency) as well as those with persistent refractory cardiac injury, with or without hemodynamic instability, and who had not responded to treatment (emergency/rescue), were excluded. These exclusion criteria were designed to ensure cohort homogeneity. By focusing on elective procedures, the study minimized the confounding effects of preoperative hemodynamic instability. This enables a more rigorous assessment of how operative timing specifically influences outcomes in stable RHD patients.

### 2.2 Study design

The patients were divided into two groups based on operation start time: the morning surgery group (AM group, 7 a.m. to 1 p.m.) and the afternoon surgery group (PM group, after 1 p.m.). To isolate the impact of start time, cases starting before 1 p.m. remained in the AM group regardless of the total procedure duration. All patients underwent conventional thoracotomy. The time at which the skin incision was made was considered the initial exposure time. We evaluated 13 preoperative and five operative patient characteristics (Tables 1, 2), along with eight endpoints (Table 3). Patient preoperative characteristics included age, sex, hypertension, diabetes, coronary artery disease, secondary pulmonary hypertension, chronic lung disease, peripheral vascular disease, renal insufficiency, liver insufficiency, and cardiac function. All procedures were performed by the same surgical team using standardized protocols. A rotational shift system for anesthesia and nursing staff was implemented to minimize fatigue and ensure consistent perioperative care quality regardless of start time.

**Table 1:** Patient characteristics before and after propensity score matching.

Characteristic	Original simple characteristics			Propensity score matched		
	AM group *N* = 147	PM group *N* = 153	SB (%)	AM group *N* = 137	PM group *N* = 146	SB (%)
Age, year	51 ± 9.5	51 ± 9.7	9.9	51 ± 9.6	51 ± 9.8	8.5
Gender, *n* (%male)	61 (41.5)	58 (37.9)	−7.3	56 (41.61)	56 (39.04)	3.9
Weight, kg	54 ± 9.3	56 ± 10.1	−20.4	55 ± 9.3	56 ± 10.	2.9
Hypertension, *n* (%)	10 (7.48)	13 (9.15)	−6.0	11 (8.03)	13 (8.90)	6.8
Diabetes, *n* (%)	6 (4.08)	2 (1.31)	17.1	3 (2.19)	2 (1.37)	−9.0
NYHA II/III/IV	3.1 ± 0.35	3.1 ± 0.43	−7.2	3 ± 0.3	3 ± 0.4	−1.1
AF, *n* (%)	74 (50.34)	82 (53.59)	−6.5	72 (52.55)	78 (53.42)	−3.0
Chronic lung disease, *n* (%)	31 (21.09)	19 (12.41)	23.3	27 (19.71)	19 (13.01)	0.8
PHD, *n* (%)	53 (36.05)	31 (20.26)	35.5	44 (32.12)	31 (21.23)	2.3
PVD, *n* (%)	5 (3.40)	10 (6.53)	−14.4	5 (3.65)	6 (4.11)	−1.2
CAD, *n* (%)	28 (19.04)	32 (20.91)	−4.7	25 (18.25)	29 (19.86)	2.6
Renal insufficiency, *n* (%)	25 (17.00)	19 (12.41)	12.9	22 (16.06)	19 (13.01)	3.0
Liver insufficiency *n* (%)	28 (19.04)	35 (22.88)	−9.4	28 (20.44)	32 (21.92)	1.8

PVD, peripheral vascular disease; PHD, pulmonary hypertension disease; CAD, coronary artery disease; NYHA, New York Heart Association functional class; AF, atrial fibrillation; SB, standardized bias.

SB assesses the balance of a measured covariate between comparison groups in a propensity-matched analysis. A covariate is considered well balanced if the SB is <10%.

**Table 2:** Operative characteristics.

Characteristic	Original simple characteristics			Propensity score matched		
	AM group	PM group	SB (%)	AM group	PM group	SB (%)
Surgeon			10			−2.5
1, *n* (%)	44 (29.93)	35 (34.64)		41 (29.93)	47 (32.19)	
2, *n* (%)	103 (70.07)	100 (65.36)		96 (70.07)	99 (67.81)	
Surgical approach			−4.2			0
MVR, *n* (%)	115 (78.23)	117 (76.47)		109 (79.56)	113 (70.40)	
DVR, *n* (%)	32 (21.77)	36 (23.53)		28 (20.44)	33 (22.60)	
Surgery time (min)	241 ± 70.1	246 ± 69.2	7.2	241 ± 71.2	246 ± 69.8	3.0
Intraoperative cardiopulmonary bypass time (min)	121 ± 55.7	120 ± 42.9	2.9	120 ± 56.0	119 ± 43	9.2
Aortic cross-clamp time (min)	97 ± 43.1	89 ± 33.2	6.4	90 ± 43.2	89 ± 33.4	9.6

MVR, mitral valve replacement; DVR, double valve replacement.

**Table 3:** Postoperative outcomes for the propensity-matched sample.

Outcome	AM group	PM group	*P-*value	OR (95% CI)
Operative mortality [*n* (%)]	3 (0.02)	8 (0.05)	0.17	0.31 (0.06–1.63)
Reoperation for bleeding [*n* (%)]	6 (0.04)	6 (0.04)	0.70	1.30 (0.33–5.09)
Postoperative transfusion (µ)	RED:2.0 ± 3.0	RED:2.0 ± 3.0	RED:0.96	0.99 (0.88–1.11)
	FFP:2.0 ± 2.2	FFP:2.0 ± 4.2	FFP:0.61	0.96 (0.86–1.09)
Total ventilation hours	19 ± 26.1	20 ± 31.6	0.37	0.99 (0.97–1.01)
ICU length of stay (h)	37 ± 33.1	35 ± 44.5	0.12	1.01 (0.99–1.02)
Hospital length of stay (days)	29 ± 12.4	27 ± 10	0.01*	1.05 (1.01–1.09)
Total hospitalization cost	108,344 ± 30,469.39	108,276.4 ± 36,498.16	0.40	0.99 (0.99–1.00)
Postoperative length of stay (days)	15 ± 8.01	15 ± 7.41	0.12	0.96 (0.91–1.01)

OR, odds ratios; CI, confidence interval.

* *P* < 0.05.

### 2.3 Endpoints

The primary endpoint of the study was operative mortality (defined as all-cause death within 30 days of surgery or during the index hospitalization), with the secondary endpoint being postoperative complications such as bleeding necessitating re-thoracotomy for hemostasis, deep chest incision infection necessitating re-debridement surgery, total perioperative blood product usage, prolonged postoperative ventilation time, intensive care unit (ICU) stay duration, total hospitalization time, and total hospitalization cost. These complications are potentially associated with surgery. Therefore, data on surgical treatment time, surgical method, surgeon, total intraoperative cardiopulmonary bypass time, and aortic cross-clamp time were considered influencing factors. All the patient variables and outcome data were collected from our institution's case management database.

### 2.4 Statistical analysis

**Figure 1:**
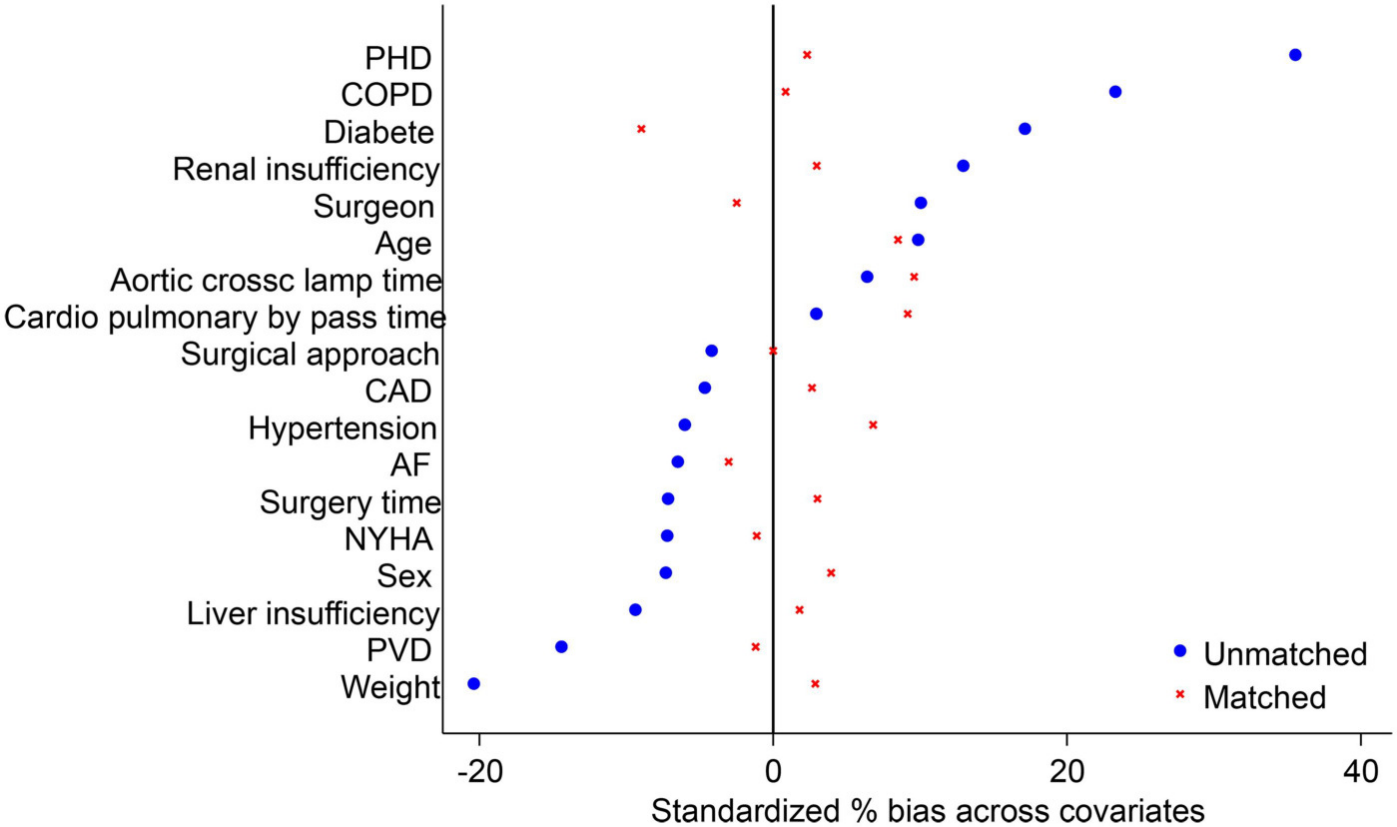
Standardized percentage bias across covariates. Love plot showing covariate balance before and after propensity score matching. Covariates were considered well balanced if the absolute value of the standardized deviation was <10%. PHD, pulmonary hypertension disease; CAD, coronary artery disease; AF, atrial fibrillation; NYHA, New York Heart Association; PVD, peripheral vascular disease.

**Figure 2:**
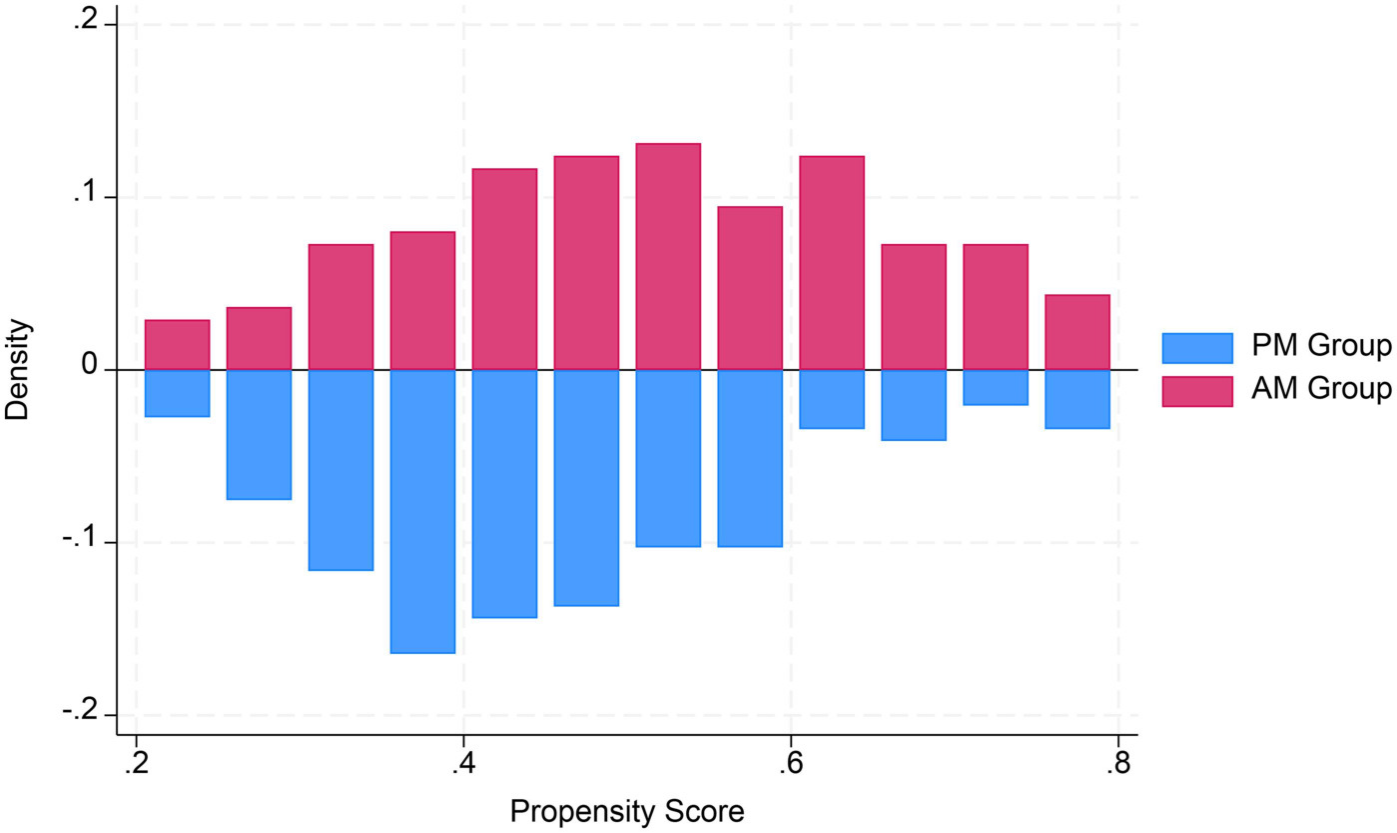
Propensity score. Mirror histograms showing propensity score distributions for the AM group and the PM group.

Data were summarized using descriptive statistics, such as percentages for categorical variables and medians (minimum, maximum) or (means ± SD) for continuous variables. Comparisons between the AM and PM groups were performed using the chi-square test or Fisher's exact test for categorical variables and Student's t test or the Wilcoxon rank-sum test for continuous variables. To minimize the influence of potential confounding factors, 1:1 propensity score matching (PSM) was performed using a logistic regression model. The matching covariates encompassed preoperative clinical status (age, sex, weight, NYHA cardiac function class, and liver/renal insufficiency), surgical complexity [specific surgical approach: MVR (mitral valve replacement) or DVR (double valve replacement)], and operator-related factors (the specific surgeon involved). For all the covariates listed in Tables 1, 2, matched PS analysis was utilized to evaluate treatment effects and multivariable logistic regression was used to estimate the PS. The dependent variable in the regression model was operation state time, which was matched with log-transformed PS at a 1:2 ratio. To achieve this, the best matching algorithm with a radius caliper width of 0.05 standard deviations was applied. Covariate balance after pairing was assessed using standardized bias, with balance considered good if the absolute value of standardized bias was less than 10%. Good matches were further illustrated by mirror histograms and love plots (Figures 1, 2). A univariate logistic regression model was used to analyze postoperative mortality and complication rates. Odds ratios were used to evaluate the advantages, with the 95% confidence intervals representing the ratio. In addition, a multivariable linear regression model was used to compare the factors influencing the total length of stay in the entire cohort, with *β* coefficients presented alongside standard errors. All analyses were performed using Stata version 18 (14). A *p*-value < 0.05 was considered statistically significant.

## 3 Results

A total of 300 patients treated during the study period were included in this retrospective analysis. All patients met the diagnostic criteria, underwent elective surgical treatment, and received valve replacement during the operation. Table 1 summarizes the general characteristics, comorbidities, and complications of both patient groups, along with a comparison of data from the two propensity score-matched groups. Before propensity score matching, there were 147 patients in the AM group and 153 patients in the PM group. After the matching process, the AM group included 137 patients, whereas the PM group included 146 patients. Among the basic preoperative characteristics of the patients, there were no significant differences in age, sex, hypertension, cardiac function, atrial fibrillation, coronary heart disease, or liver function. However, variations in weight, diabetes, pulmonary function, pulmonary hypertension, peripheral vascular disease, and renal function were observed. After matching, all the preoperative characteristics were well matched. There were no significant differences in intraoperative factors, such as surgical methods, operation time, intraoperative cardiopulmonary bypass time, or aortic cross-clamping time. Although there was slight variation in the surgeon involved, the results after matching indicated satisfactory alignment. There were no significant differences in postoperative matching results, postoperative mortality, reoperation rate due to bleeding, postoperative blood transfusion, ventilator ventilation time, ICU stay time, postoperative hospital stay, or total hospitalization cost. However, a statistically significant difference was observed in total hospitalization time.

Analysis of risk factors for total hospitalization time revealed significant effects of sex, cardiac function, peripheral vascular disease, liver function, surgical method, and postoperative hospitalization time. Notably, women had significantly shorter hospitalization times compared with men. Moreover, poorer cardiac function was associated with longer hospital stays, whereas the presence of peripheral vascular disease tended to result in shorter hospitalization periods. Liver insufficiency notably impacted the length of hospitalization, leading to a significant extension. In addition, DVR surgery, being a more complex surgical method, was associated with a more pronounced loss of cardiac function, prolonged postoperative recovery time, and extended hospitalization following surgery, collectively contributing to overall lengthening of total hospitalization time. Details of the multivariable regression coefficients and associated risk factors are presented in Table 4.

**Table 4:** Multivariable risk factors associated with hospital length of stay for the whole cohort.

Risk factors	*β* ^a^	SE	*P*	CI
Gender	−2.65	0.96	<0.01	−4.5 to −0.75
NYHA	3.39	1.23	<0.01	0.96–5.8
PVD	−4.9	2.24	0.02	−9.35 to −0.49
Liver insufficiency	2.47	1.18	0.03	0.15–4.80
Surgical approach				
1. MVR	Reference			
2. DVR	4.84	1.66	<0.01	1.54–8.10
Postoperative length of stay	0.97	0.06	<0.01	0.84–1.09

SE, standard error.

^a^ *β*, coefficients for the linear regression.

## 4 Discussion

In this retrospective study conducted at a single center, we analyzed operation start times in patients who were diagnosed with rheumatic heart disease who underwent elective surgical treatment. There was no significant difference in operative mortality rate or postoperative complication risk between patients who underwent surgery in the morning and those who underwent surgery after 1 p.m.

Similar studies have also suggested that surgery has no significant effect on patient prognosis. In a study on organ transplant surgery, Fechner et al. compared 260 patients who underwent kidney transplantation over 10 years. The findings indicated that patients who underwent surgery later in the day experienced a higher complication rate and were at greater risk of graft failure than those who underwent surgery earlier in the day (9). Lonze BE et al. studied 578 patients who underwent liver transplantation and determined that there were no significant differences in complication rates between daytime and evening surgeries. Nevertheless, evening surgeries were associated with longer operation times and a notable increase in mortality (9).

In cardiothoracic surgery, a comparative study was conducted by Gu-Ha and A-Lai et al. on patients who underwent lung surgery at a large surgery center over 2 months. The study revealed no significant differences in operation time, postoperative performance time, costs, or postoperative complication rates between the morning surgery group and the afternoon surgery group (10). Similarly, in heart and lung transplantation, George et al. compared outcomes in patients who underwent these procedures in the United States over 10 years. They discovered that patients who underwent surgery in the morning had similar postoperative survival and complication rates to those who underwent surgery in the afternoon or at night, with no significant differences observed (11).

In the field of cardiac surgery, Samantha Nemeth et al. investigated the effects of conducting CABG and AVR surgeries at various time points. The results revealed mixed differences in ICU hospitalization time, postoperative hospitalization time, use of blood products, and aortic occlusion time. Factors such as mortality and ventilator-assisted ventilation time were found to have no significant impact (12). Heller et al. conducted a comparative study on patients who underwent elective cardiac surgery at multiple time points over 3 years. The findings indicated that there were no statistically significant differences in postoperative mortality, length of hospital stay, or other outcomes based on the start time of elective cardiac surgery (13).

In our study, patients with rheumatic heart disease who underwent valve replacement surgery were divided into AM and PM surgery groups, and our results align with previously published findings. We observed no significant differences between the two groups in postoperative mortality rate, reoperation rate, use of blood products, ventilator-assisted ventilation time, ICU stay time, postoperative hospitalization time, or total hospitalization cost. The total length of hospital stay was the only factor observed to be different between the two groups. Notably, our propensity score matching (Table 1) confirmed that all preoperative risks and surgical complexity factors were well balanced [all standardized bias (SB) < 10%, *P* > 0.05]. This suggests that the longer stay in the AM group is likely a stochastic finding within this high-complexity RHD cohort, rather than a result of unbalanced baseline characteristics.

From a mechanistic perspective, although the circadian clock gene *Rev-Erbα* has been shown to modulate myocardial tolerance to ischemia-reperfusion injury—with some studies suggesting an “afternoon advantage”—our data did not reflect this time-of-day effect. This discrepancy likely stems from the advanced pathophysiological stage of rheumatic heart disease. In our cohort, the profound myocardial structural remodeling and chronic overload, combined with standardized modern myocardial protection protocols, may have overshadowed the subtle endogenous influences of biological rhythms. Thus, while chronobiological factors are theoretically relevant, they appear not to be a primary determinant of short-term outcomes in elective RHD valve surgery (6).

The observed median hospital stay in our cohort was longer than that typically reported in Western centers. This is primarily due to the advanced disease stage and poor nutritional status of RHD patients in Southwest China, many of whom present with severe cardiac dysfunction and secondary organ impairment. Such patients require extended preoperative optimization to mitigate surgical risks. Furthermore, due to the lack of local post-acute rehabilitation facilities, patients remain hospitalized to complete warfarin titration and physical recovery. This practice, combined with a regional healthcare model that prioritizes comprehensive in-hospital recovery for patients from geographically remote areas, contributes to the extended duration of hospital stay.

Statistical analysis was conducted on the risk factors associated with the total length of hospitalization. Factors such as sex, cardiac function status, peripheral vascular disease, liver insufficiency, surgical method, and postoperative hospitalization time were found to be related. Among these factors, men had significantly longer hospitalization times than women. A decrease in heart function was noted to be more harmful, impacting the function of the body's organs, leading to a more extended postoperative recovery period. Liver function also played a significant role, as the liver is responsible for producing and utilizing various nutrients. If liver function is inadequate, the overall recovery time for the body's organs can be prolonged. In addition, the surgical method used was a key factor, with DVR surgery being more complex and time-consuming than MVR surgery. This complexity resulted in longer surgical times, consequently extending postoperative recovery and hospitalization time. One of the only factors negatively correlated with postoperative recovery time was peripheral vascular disease (PVD). We consider that patients with PVD may undergo more extensive vascular interventions before surgery, resulting in improved heart and blood vessel function, ultimately leading to faster postoperative recovery.

This study has several limitations. First, its retrospective, single-center design and relatively small sample size from a specific region may limit the generalizability of the findings. Second, the exclusion of emergency cases might introduce selection bias, although we employed PSM to ensure well-balanced baseline characteristics. Finally, this study focused on short-term perioperative outcomes; long-term survival and quality-of-life measures were not assessed. Future multicenter studies with larger cohorts and longer follow-up are warranted to validate these results.

## 5 Conclusion

In our regression study, we analyzed the outcomes of patients with rheumatic heart disease who underwent elective heart valve surgery at different times of the day. Our results indicated that the timing of surgery, whether in the morning or afternoon, did not significantly affect postoperative mortality or reoperation rates, postoperative hospitalization time, or total hospitalization expenses. However, it did influence the total hospitalization time. Although long-term continuous work may increase the burden on medical staff, whether surgery is performed in the morning or the afternoon does not result in significant differences for patients. Therefore, patients can undergo surgical treatment at different times with confidence.

## Data Availability

The datasets generated and/or analyzed during the current study are available from the corresponding author upon reasonable request.
